# Additional Evidence for Neuropsychiatric Manifestations in Mosaic Trisomy 20: A Case Report and Brief Review

**DOI:** 10.3390/children8111030

**Published:** 2021-11-10

**Authors:** Marco Colizzi, Giulia Antolini, Laura Passarella, Valentina Rizzo, Elena Puttini, Leonardo Zoccante

**Affiliations:** 1 Child and Adolescent Neuropsychiatry Unit, Maternal-Child Integrated Care Department, Integrated University Hospital of Verona, 37126 Verona, Italy; giuliaantolini11@gmail.com (G.A.); laura.passarella17@gmail.com (L.P.); valentinaannarizzo@gmail.com (V.R.); elena.puttini@aovr.veneto.it (E.P.); leonardo.zoccante@aovr.veneto.it (L.Z.); 2 Section of Psychiatry, Department of Neurosciences, Biomedicine and Movement Sciences, University of Verona, 37134 Verona, Italy; 3 Department of Psychosis Studies, Institute of Psychiatry, Psychology and Neuroscience, King’s College London, London SE5 8AF, UK

**Keywords:** pediatric conditions, psychiatric genetics, anger, self-regulation, aneuploidy, autosomal trisomy

## Abstract

Mosaic trisomy 20 is a genetic condition in which three chromosomes 20 are found in some cells. Its clinical phenotype seems to be highly variable, with most features not reported across all individuals and not considered pathognomonic of the condition. Limited and recent evidence indicates that neuropsychiatric manifestations may be more present in the context of trisomy 20 than was once thought. Here, we present a case of a 14-year-old female adolescent of White/Caucasian ethnicity with mosaic trisomy 20, who was admitted twice to an inpatient Child and Adolescent Neuropsychiatry Unit for persisting self-injury and suicidal ideation. A severe and complex neuropsychiatric presentation emerged at the cognitive, emotional, and behavioral levels, including mild neurodevelopmental issues, isolation, socio-relational difficulties, depressed mood, temper outbursts, irritability, low self-esteem, lack of interest, social anxiety, panic attacks, self-cutting, and low-average-range and heterogeneous intelligence quotient profile. Particularly, the patient was considered at high risk of causing harm, mainly to self, and appeared to be only partially responsive to medication, even when polypharmacy was attempted to improve clinical response. Except for school bullying, no other severe environmental risk factors were present in the patient’s history. The patient received a diagnosis of disruptive mood dysregulation disorder.

## 1. Introduction

Aneuploidy in natural conception is an estimated 0.3% [1], with 0.6 per 10,000 births presenting a rare mosaic trisomy [2] and trisomy 20 accounting for 16% of all mosaicisms [3]. Trisomy 20 is a genetic condition caused by an extra chromosome at position 20. While complete trisomy 20 is rare and suggested not to be compatible with life, a mosaic form of trisomy 20, where three chromosomes 20 are found only in some cells, may be possible, and thought to result in a grossly normal phenotype in most of the cases [4]. However, a recent review of the scientific literature revealed a paucity of studies reporting the phenotypic manifestations potentially associated with the condition [5]. To date, the clinical phenotype of trisomy 20 seems to be highly variable. In fact, most features are not reported across all individuals and are not considered pathognomonic of the condition, as they are also found in the general population, although at a lower rate [4]. More specifically, a number of craniofacial [4,6,7,8,9,10,11,12,13], cutaneous [11,14,15], cardiovascular-pulmonary [7,16,17,18,19], gastrointestinal [20], endocrinological [15], reproductive [10,15], locomotor [4,7,13,16,20,21,22], nervous [4,10,14,19,21,23], and cognitive and mental [4,7,13,15,18,20,22,23,24,25] features have been reported.

Recent evidence indicates that neuropsychiatric manifestations may be more present in the context of trisomy 20 than was once thought, with also potential forensic implications [5]. So far, it has been suggested that children with trisomy 20 may present with social [20], emotional [20], and learning [20,22] deficits, as per altered neurodevelopment [20,24]. Less clear is the persistence of psychiatric and behavioral features through adolescence and early adulthood.

Here, we report the case of a 14-year-old female adolescent of White/Caucasian ethnicity with mosaic trisomy 20, who was admitted twice to the inpatient Child and Adolescent Neuropsychiatry Unit of the Integrated University Hospital of Verona for persisting self-injury and suicidal ideation. Such admissions followed a neuropsychiatric assessment in the community and a presentation to the Accident & Emergency (A&E) Department, respectively. These were her first presentations to a neuropsychiatric ward. Her developmental history, neuropsychiatric presentation at admission, diagnosis, and clinical evolution are presented in detail in the following sections.

## 2. Case Report

### 2.1. Medical History

**Figure 1:**
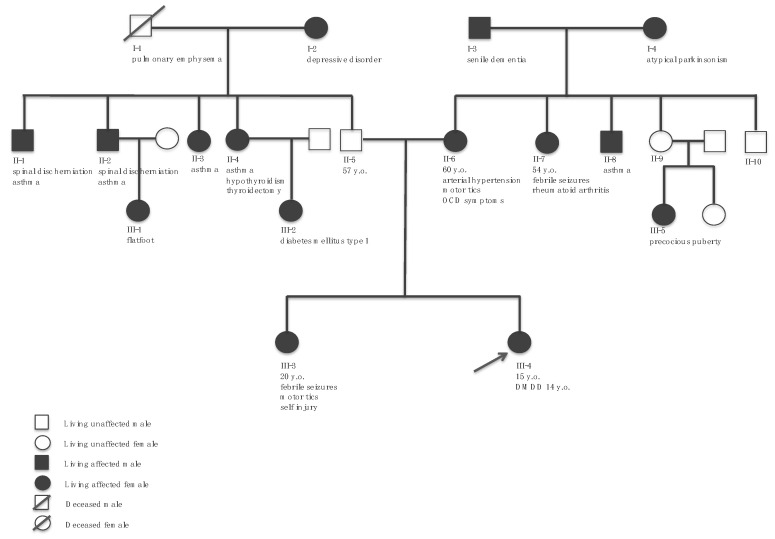
Pedigree of the patient. DMDD, disruptive mood dysregulation disorder.

A detailed medical history was obtained from the patient and her parents. She was raised by her mother and father and currently lives at home with both her parents and her only sibling, an older sister. Her family history is positive for depressive disorder in her paternal grandmother, obsessive-compulsive disorder symptoms in her mother, self-injury in her sister, motor tics (self-limited childhood tics, including eye-winking movements as well as hand and finger twitching/stretching) in her mother and sister, and epileptic manifestations in her sister, maternal aunt, and paternal second cousin. A family history of joint laxity and hernias was also reported. As the patient’s sister was not considered to have a clinically detectable mosaicism, formal genetic testing has never been performed, also considering the difficulties of ascertaining low-level mosaicism where most cell lineages are not affected. A full pedigree providing a graphic depiction of the patient’s family structure and medical history is reported in Figure 1.

She was born to term out of her parents’ third pregnancy (the first ended in a spontaneous abortion). The pregnancy was uncomplicated, but amniocentesis, performed due to advanced maternal age, showed a karyotype of 47,XX + 20/46,XX in 10 of 32 metaphases analyzed. Her Apgar score, birth weight, length, and head circumference were all within normal ranges. Successively performed abdominal ultrasound and transfontanelle ultrasonography turned out negative. Overall, her development milestones proceeded regularly and on time, except for a slight delay in gross and fine motor skills. Starting from the pediatric age, physical examination findings related to her genetic condition included a broad and depressed nasal bone, low-set ears, hypotonia, and joint laxity. Physiatric assessment also highlighted the presence of flatfoot, a slight degree of shoulders asymmetry, and left convex scoliosis. During the years of primary school, facial tics and compulsive repetitive behaviors made their first appearance, together with the findings of a slight degree of dysgraphia and astigmatism. In secondary school, following episodes of bullying and cyberbullying, a progressive decline in her school performance was observed, along with the tendency to isolate herself from others, with difficulties in building relationships with peers, resulting in a poor friendship network. Furthermore, a continuous depressed mood throughout the day, associated with temper outbursts and irritability (in the absence of clear mood swings), low self-esteem, a lack of interest in any activity, significant social anxiety, panic attacks, daily episodes of self-cutting (occurring both at school and at home), and suicidal ideation were reported. Such symptoms did not appear to negatively affect the sleep–wake cycle.

Due to her neuropsychiatric distress, the patient was referred to the Childhood Neuropsychiatry Territorial Service, where a psychotherapeutic intervention was implemented, along with pharmacological treatment consisting of sertraline oral solution 20 mg/mL, at a dosage of 1.25 mL per day, and alprazolam 0.75 mg/mL oral concentrate, at a dosage of 0.125 mg per day (5 drops). Such therapy did not bring any benefit to the patient. Therefore, it was decided to admit her to the inpatient Child and Adolescent Neuropsychiatry Unit.

### 2.2. Clinical Course, Diagnostic Conclusions, and Outpatient Follow-Up

During hospitalization, she maintained overall good health and the following clinical evaluations were performed, aimed at monitoring her at both the clinical and the therapeutic level: COVID-19 nasal swab testing prior to admission, routine blood exams, urinalysis, and electrocardiogram; all of these turned out negative. Based on preexisting documentation and direct clinical observation, information was gathered with reference to her trisomy 20 manifestations. A detailed report of signs and symptoms, as contrasted to what is to be expected based on the current literature, is provided in Table 1.

A psychological assessment was also carried out using the Child Behavior Checklist (CBCL) 6–18 [26] compiled by both the patient and her mother, who was with her throughout the hospitalization. CBCL subscales suggesting pathological symptoms (scores > 70) were “Anxious/Depressed”, “Withdrawn/Depressed”, “Social Problems”, “Thought Problems”, “Attention Problems”, “Depressive Problems”, “Anxiety Problems”, “Sluggish Cognitive Tempo”, and “Stress Problems”. “Somatic Complaints” and “Obsessive-Compulsive Problems” were borderline symptoms according to her mother, while being rated as pathological by the patient (Table 2).

Throughout her hospital stay, the patient remained disheveled and unkempt. Self-cutting injuries were evident on her hands and neck, and upon further examination, they covered most of the forearms and thighs. The injuries were shallow and appeared to be in multiple stages of healing. She wore loose-fitting clothing, mostly in black. During interviews, she spoke reticently and only after requesting that her mother left the room. She appeared tense and had difficulty sustaining eye contact. Her speech was scarce, low in volume, and lacking modulation. She only answered questions when prodded, and her replies lacked articulation. She was coherent and oriented to person, place, and time. There were no signs of perceptual abnormalities. Hallucinations, depersonalization, derealization, and dissociative phenomena were denied. The patient did not show any loosening of association, flight of ideas, tangentiality, or circumstantiality. Her affect was flat but not inappropriate to the content of her speech, which was focused on the circumstances of her hospitalization, her emotional malaise, and her continued self-harm ideation. Some anxiety through repetitive wringing of her hands was observed. She reported that she had no appetite but had no trouble sleeping at night.

Considering the suboptimal symptom control, a change in pharmacological treatment was made. In particular, sertraline and alprazolam were discontinued, whereas pregabalin at a dosage of 150 mg, fluvoxamine at a dosage of 50 mg, and quetiapine extended release at a dosage of 50 mg were initiated. The patient received a diagnosis of disruptive mood dysregulation disorder (DMDD), according to the Diagnostic and Statistical Manual of Mental Disorders, -Fifth Edition DSM-5 [27], and was discharged 7 days later in good clinical condition, with the indication to continue the treatment with pregabalin, fluvoxamine, and quetiapine.

At a follow-up visit, planned 1 week later, the patient presented with symptoms of a potential relapse. Thus, the dosages of fluvoxamine and quetiapine extended release were increased to 75 mg and 150 mg, respectively. However, after 1 more week, she entered the A&E Department presenting with the same symptoms she had when she was admitted for the first time, namely remarkable social anxiety and panic attacks, significantly low mood, daily episodes of self-injury, and suicidal ideation. Using a pencil sharpener blade, she had cut her forearms, abdomen, and legs, to the point where she had carved the word “help” into the skin of her left thigh. The neuropsychiatrist assessment advised for another hospitalization, and she was hence admitted after a negative COVID-19 nasal swab. Besides multiple routine blood exams and electrocardiograms, all of which turned out negative, this time a plastic surgical evaluation and a cardiological assessment with color-Doppler ultrasound were also performed, which showed nothing pathological.

Despite the initial improvement, as the pharmacological treatment had not led to clinical stability, it was modified as follows: pregabalin and quetiapine were discontinued, whereas lithium prolonged release at a dosage of 166 mg per day and olanzapine at a dosage of 10 mg per day were introduced. Moreover, the fluvoxamine dosage was increased to 150 mg. The previous diagnosis of disruptive mood dysregulation disorder (DMDD), according to DSM-5, was confirmed, and she was discharged 13 days later in good overall clinical condition, with the indication to continue the treatment with lithium prolonged release, fluvoxamine, and olanzapine.

A post-discharge assessment performed 2 weeks later found the patient overall clinically stable but only partially responsive to treatment. Despite improvement, her mood was still oriented toward depression and socio-emotional difficulties were still reported, resulting in outbursts and irritability. An abdominal ultrasound was also performed, which turned out negative, with liver, hepatic ducts, portal vein, gallbladder, kidneys, spleen, pancreas, and bladder all normal in appearance and echotexture. Furthermore, after about 4 weeks of pharmacological treatment stability, a neurocognitive assessment was performed using the Wechsler Intelligence Scale for Children–Fourth Edition (WISC-IV) [28]. The patient’s full-scale intelligence quotient (FSIQ) was in the low average range as for normative data (FSIQ = 80), and a heterogeneous profile emerged, with “Perceptual Reasoning” falling in the average range, “Processing Speed” in the low average range, and “Verbal Comprehension” and “Working Memory” in the very low range (Table 2).

## 3. Discussion

The past decades have witnessed an increasing interest in the role of genetics in shaping human behavior. Genome-wide association studies (GWASs) have helped detect the polygenic architecture of psychiatric disorders, with potential applications in clinical settings [29]. Disruptive mood dysregulation disorder (DMDD) is no exception. Since its introduction in the DSM-5 [27], studies have investigated the role of genetics in modulating emotional and behavioral functioning in youth suffering from the condition. Research interest has focused on genetic variation affecting the function of neurobiological systems, such as the opioid system, that are known to modulate cognitive, emotional, and social behaviors [30]. In addition, studies support developmentally dynamic genetic effects on core features of the disorder, such as irritability, with genetic influences being higher in the female gender and as compared to environmental features [31]. However, the environment is still believed to exert a modulatory effect [32]. Consistently, evidence from gene-by-environment-interaction studies suggests that genetic variation in the hypothalamic–pituitary–adrenal (HPA) axis may interact with the effect of chronic stress in increasing the risk of presenting with a negative affect [33]. The latter is considered one of the factors predicting later psychopathology [34] as well as stability of DMDD symptoms, such as an irritable–angry mood and temper outbursts, through adolescence and early adulthood.

The first linkage study of psychosis using empirically derived, clinically homogeneous phenotypes, defined by symptomatic profiles rather than operationalized diagnostic criteria, revealed an unexpected linkage to chromosome 20 for “Schizomania” and “Mania” latent classes of psychotic illness as well as the highest logarithm of the odds (LOD) score of any latent class in the genome for the “Deficit Syndrome” class [35]. That chromosome 20 may be involved in both manic/positive and depressive/negative symptoms of psychosis is supported by genome scan studies and meta-analyses finding evidence of linkage for bipolar disorder and schizophrenia [35,36,37,38,39,40], as well as a comprehensive gene-based association study of 327 genes on chromosome 20, identifying two loci, *R3HDML* and *C20orf39*, associated with depressive symptoms in psychotic illness [41]. Taken together, these studies are compelling in their support of chromosome 20 harboring genes relevant to the affective component of schizophrenia and conditions presenting with admixtures of mood and psychotic symptoms [42]. Further, chromosome 20 has been associated with affective psychoses characterized by suicidal attempts [40], and a recent genome-wide association study identified a number of single-nucleotide polymorphisms (SNPs), all on chromosome 20, supporting a genetic transmission of suicide attempts, not entirely accounted for by suffering from a mental disorder [43]. Interestingly, functional SNPs on chromosome 20 influencing gene expression and bipolar disorder susceptibility have been shown to modulate hippocampal volume and cognitive performance in healthy individuals [44].

Along with numerical chromosome anomalies, such as mosaicism, structural anomalies of the chromosome 20, including ring chromosome [45], deletion of the short arm [46], microduplication within the short arm [47], and microdeleletion of the long arm [48], have also been associated with alterations in several body systems and functions as well as neurocognitive difficulties and neuropsychiatric features. Commonly reported behavioral manifestations include developmental delay, intellectual disability, sensory processing disorder, poor motor coordination, impaired speech and executive abilities, apathy or hyperactivity, loss of social skills and poor emotional regulation, obsessive behavior, psychosis, and autistic features.

Of further interest, the activity-dependent neuroprotective protein (ADNP) syndrome, which was only discovered in 2014, is a neurodevelopmental genetic disorder caused by changes (mostly de novo mutations) in the *ADNP* gene, which is located on the long arm of chromosome 20. It is frequently associated with developmental delays, intellectual delays, motor planning delays, delayed or absent speech, and autism features of varying degrees [49]. A potential role in self-injurious behavior has also been suggested [50]. Multiple body systems may be affected, including the brain (e.g., developmental delay, intellectual disability), heart (e.g., atrial septal defect, patent ductus arteriosus), immune system (e.g., frequent infections), gastrointestinal system (e.g., gastroesophageal reflux, constipation), endocrine system (e.g., early puberty, thyroid hormone problems), and musculoskeletal system (e.g., joint hypermobility, scoliosis) [49]. Finally, several pathogenic and possibly pathogenic variants in the coding region of the prion protein gene (*PRNP*), which is also located on chromosome 20, have been associated with different clinical phenotypes of neurodegenerative diseases, including Creutzfeldt–Jakob disease, Gerstmann–Straüssler–Scheinker disease, fatal familial insomnia, and other types of dementia [51].

In summary, increasing evidence from studies of the numerical and structural variation of the genome at the chromosomal and subchromosomal levels supports a role of chromosome 20 in the manifestation of a wide range of neuropsychiatric features. A recent case report on a young adult male individual with trisomy 20 reported some novel neuropsychiatric symptoms that had never been associated with the condition, such as childhood-onset visual hallucinations and self-injury, including biting self and headbanging, hypothesizing an underestimation of the extent of mental disorders in the context of trisomy 20 [5]. In addition, authors suggested that several environmental risk factors encountered in their case, such as child abuse, family discord, and exposure to domestic violence, may have at least partially accounted for the unusually severe psychiatric presentation as compared to previously reported cases, where, instead, milder or subthreshold psychiatric symptoms may have been overlooked [5]. Noteworthy, in that case, trisomy 20 was successfully applied as a mitigating factor in a capital murder trial [5].

The current trisomy 20 case revealed a severe and complex neuropsychiatric presentation at the cognitive, emotional, and behavioral levels. In addition, the patient was considered at high risk of causing harm, mainly to self, and appeared to be only partially responsive to medication, even when polypharmacy was attempted to improve clinical response. To the best of our knowledge, except for school bullying and the potentially stressful impact of the sister’s self-harm, no other severe environmental risk factors were present in the patient’s history.

Due to mosaicism, a remarkable variability in clinical symptoms between cases is not surprising. It is plausible to hypothesize that depending on the degree of mosaicism, body systems may be differentially affected, with implications for the severity of the phenotypic expression. Further, the potential correlation between the level of trisomic cells and clinical outcome is currently unknown and deserves investigation, as for other chromosomal mosaicisms [52]. In fact, it is likely that patients with trisomy 20 presenting with more evident neuropsychiatric distress have a higher percentage of affected cells in the brain as well as systems other than the central nervous system (CNS), such as the immune, cardiometabolic, and hypothalamic–pituitary–adrenal (HPA) systems, which have been involved in the onset of major psychiatric disorders [53].

## Figures and Tables

**Table 1:** Trisomy 20 manifestations in the current case as compared to the previous literature.

Body Systems and Functions	Symptoms/Signs	Current Case + Present X Not Present
Craniofacial		
Korkontzelos (2017) [6], Velissariou et al. (2002) [7]	Underdeveloped nasal bone	+
Hsu et al. (1991) [4], Mavromatidis et al. (2010) [8], Myers and Prouty (1989) [9], Ensenauer et al. (2005) [10]	Ear morphology abnormalities	+
Velissariou et al. (2002) [7], Warren et al. (2001) [11]	Micrognathia and retrognathia	X
Stromme et al. (2005) [12]	Cleft lip and palate	X
Hsu et al. (1991) [4], Velissariou et al. (2002) [7], Reish et al. (1998) [13]	Abnormal periorbital region morphology	X
Cutaneous		
Powis and Erickson (2009) [14]	Thin and brittle nails	X
Warren et al. (2001) [11], Powis and Erickson (2009) [14], Girard et al. (2005) [15]	Hypomelanosis of Ito	X
Hartmann et al. (2004) [18]	Linear and whorled nevoid hypermelanosis	X
Velissariou et al. (2002) [7]	Mongolian spots	X
Cardiovascular-Pulmonary		
Morales et al. (2010) [16]	Pulmonary isomerism	X
Velissariou et al. (2002) [7], Karaoguz et al. (2007) [17], Hartmann et al. (2004) [18], Hsieh et al. (1992) [19]	Congenital heart defects	X
Gastrointestinal		
Willis et al. (2008) [20]	Stipsis	+
Endocrinological		
Girard et al. (2005) [15]	Growth hormone deficiency	X
Reproductive		
Ensenauer et al. (2005) [10], Girard et al. (2005) [15]	Cryptorchidism	X
Locomotor		
Willis et al. (2008) [20], Stein et al. (2008) [21]	Sloped shoulders	+
Velissariou et al. (2002) [7], Willis et al. (2008) [20]	Abnormal spinal column morphology	+ (Scoliosis)
Velissariou et al. (2002) [7], Reish et al. (1998) [13], Willis et al. (2008) [20], Stein et al. (2008) [21], Holzgreve et al. (1986) [22]	Central and peripheral altered muscle tone	+ (Hypotonia)
Velissariou et al. (2002) [7]	Ligamentous laxity	+
Morales et al. (2010) [16]	Camptodactyly	X
Hsu et al. (1991) [4], Velissariou et al. (2002) [7]	Clinodactyly	X
Montplaisir (2019) [5]	Polydactyly	X
Stein et al. (2008) [21]	Rib anomalies	X
Nervous		
Powis and Erickson (2009) [14]	Epileptic manifestations	X
Ensenauer et al. (2005) [10]	Hearing impairment	X
Hsu et al. (1991) [4], Salafsky et al. (2001) [23]	Microcephaly	X
Hsieh et al. (1992) [19], Stein et al. (2008) [21]	Abnormal neuroimaging findings	X
Cognitive and Mental		
Willis et al. (2008) [20]	Preserved IQ and learning disabilities	+
	Repetitive behaviors	+
	Isolation	+
	Social and emotional difficulties	+
	Pragmatic/Social communication difficulties	+
Wallerstein et al. (2005) [24]	Atypical neurodevelopment with attention deficit	X
	Impulsivity	+
	Atypical social reciprocity	+
Girard et al. (2005) [15]	Speech difficulties	X
Hsu et al. (1991) [4], Velissariou et al. (2002) [7], Girard et al. (2005) [15], Hartmann et al. (2004) [18], Salafsky et al. (2001) [23], Miny et al. (1989) [25]	Developmental psychomotor difficulties	+
Reish et al. (1998) [13]	Impaired fine and gross motor abilities	+
Holzgreve et al. (1986) [22]	Developmental language difficulties	X
Montplaisir (2019) [5]	Self-injury	+
Montplaisir (2019) [5]	Hallucinations	X

**Table 2:** Neurocognitive and psychological assessment.

Neurocognitive Assessment			
Wechsler Intelligence Scale for Children–Fourth Edition (WISC-IV)	Score (95% Confidence Interval; subtest raw score)		
Full-Scale Intelligence Quotient	80 (75–87)		
Verbal Comprehension	78 (72–86; Similarities: 7; Comprehension: 3; Vocabulary: 7; Information (supplemental subtest): 5)		
Perceptual Reasoning	95 (87–103; Block Design: 11; Matrix Reasoning: 9; Picture Concepts: 8)		
Working Memory	79 (72–90; Subtest: Digit Span: 7; Letter–Number Sequencing: 6; Arithmetic (supplemental subtest): 3)		
Processing Speed	85 (77–97; Coding: 8; Symbol Search: 7)		
**Psychological Assessment**			
Child Behavior Checklist (CBCL)	T-Scores (Patient)	T-Scores (Mother)	Clinical: T ≥ 70 Borderline: 65 ≥ T < 70 Non-clinical: T < 65
Syndrome Scale Scores			
Social Problems	70	70	Clinical
Thought Problems	73	73	Clinical
Attention Problems	83	70	Clinical
Internalizing Problems			
Withdrawn/Depressed	100	100	Clinical
Anxious/Depressed	100	74	Clinical
Somatic Complaints	70	65	Clinical/Borderline
Externalizing Problems			
Rule-Breaking Behavior	60	57	Non-clinical
Aggressive Behavior	58	58	Non-clinical
Total Problems			
Internalizing Score	86	77	Clinical
Externalizing Score	59	58	Non-clinical
Total Problems Score	74	70	Clinical
DSM-Oriented Scales			
Depressive Problems	88	79	Clinical
Anxiety Problems	94	73	Clinical
Somatic Problems	59	59	Non-clinical
Attention Deficit	69	60	Borderline/Non-clinical
Oppositional Defiant Problems	59	63	Non-clinical
Conduct Problems	61	55	Non-clinical
Other Scales			
Sluggish Cognitive Tempo	80	73	Clinical
Obsessive-Compulsive Problems	87	66	Clinical/Borderline
Stress Problems	86	78	Clinical

## Data Availability

The data reported in this paper are available from the medical history of the patient.
